# Comparison of the Diagnostic Accuracy of Five Cognitive Screening Tests for Diagnosing Mild Cognitive Impairment in Patients Consulting for Memory Loss

**DOI:** 10.3390/jcm13164695

**Published:** 2024-08-09

**Authors:** María Valles-Salgado, Jordi A. Matias-Guiu, Alfonso Delgado-Álvarez, Cristina Delgado-Alonso, María José Gil-Moreno, Esther Valiente-Gordillo, Juan Ignacio López-Carbonero, Lucía Fernández-Romero, Lidia Peña-DeDiego, Silvia Oliver-Mas, Jorge Matías-Guiu, Maria Diez-Cirarda

**Affiliations:** 1 Department of Neurology, San Carlos Institute for Health Research (IdISSC), Universidad Complutense de Madrid, 28040 Madrid, Spain; dunadelsahara@hotmail.com (M.V.-S.); jordi.matias-guiu@salud.madrid.org (J.A.M.-G.); matiasguiu@gmail.com (J.M.-G.); 2 Department of Psychobiology & Behavioral Sciences Methods, Universidad Complutense de Madrid, 28040 Madrid, Spain

**Keywords:** Addenbrooke’s Cognitive Examination, Memory Impairment Screen, mild cognitive impairment, Mini-Mental State Examination, Montreal Cognitive Assessment, neuropsychological assessment, screening, Rowland Universal Dementia Assessment Scale

## Abstract

**Objectives**: We aimed to evaluate and compare the diagnostic capacity of five cognitive screening tests for the diagnosis of mild cognitive impairment (MCI) in patients consulting by memory loss. **Methods**: A cross-sectional study involving 140 participants with a mean age of 74.42 ± 7.60 years, 87 (62.14%) women. Patients were classified as MCI or cognitively unimpaired according to a comprehensive neuropsychological battery. The diagnostic properties of the following screening tests were compared: Mini-Mental State Examination (MMSE), Addenbrooke’s Cognitive Examination III (ACE-III) and Mini-Addenbrooke (M-ACE), Memory Impairment Screen (MIS), Montreal Cognitive Assessment (MoCA), and Rowland Universal Dementia Assessment Scale (RUDAS). **Results**: The area under the curve (AUC) was 0.861 for the ACE-III, 0.867 for M-ACE, 0.791 for MoCA, 0.795 for MMSE, 0.731 for RUDAS, and 0.672 for MIS. For the memory components, the AUC was 0.869 for ACE-III, 0.717 for MMSE, 0.755 for MoCA, and 0.720 for RUDAS. Cronbach’s alpha was 0.827 for ACE-III, 0.505 for MMSE, 0.896 for MoCA, and 0.721 for RUDAS. Correlations with Free and Cued Selective Reminding Test were moderate with M-ACE, ACE-III, and MoCA, and moderate for the other tests. The M-ACE showed the best balance between diagnostic capacity and time of administration. **Conclusions**: ACE-III and its brief version M-ACE showed better diagnostic properties for the diagnosis of MCI than the other screening tests. MoCA and MMSE showed adequate properties, while the diagnostic capacity of MIS and RUDAS was limited.

## 1. Introduction

Neurodegenerative diseases, and especially Alzheimer’s disease (AD), are the most frequent cause of cognitive impairment [1]. The early detection of cognitive impairment is increasingly relevant, as it facilitates an accurate diagnosis, treatment interventions, and patient and caregiver support. Furthermore, the new therapies recently approved or under development for AD are targeting the earliest stages of the disease [2].

Mild cognitive impairment (MCI) is a stage characterized by a decline in episodic memory or other cognitive functions, in which patients are at higher risk of developing dementia due to Alzheimer’s disease or other causes [3]. MCI patients show abnormal cognitive functions compared with the expected performance according to age and education levels, but without significant impairment in daily living activities. Neuropsychological assessment is an essential tool in the diagnostic framework of patients with MCI. Cognitive screening tests have been recommended as a first-line assessment of patients consulting by cognitive issues [4]. Cognitive screening tests are carried out at different assistance levels, guiding the decisions to refer patients to more specialized centers (e.g., from primary care to neurology, or from general neurology to memory units) and for indicating neuroimaging or fluid biomarkers. The Mini-Mental State Examination (MMSE) is the most used cognitive screening test to assess patients [5]. However, several studies have concluded a limited capacity to detect early stages of AD and especially to detect patients with MCI [6]. In this regard, several tests have been developed and validated for detecting MCI [7,8].

Among the different cognitive screening tests, some of them have gained some popularity due to different reasons. The Montreal Cognitive Assessment (MoCA) includes some tasks to assess attention and executive function and could be more helpful in detecting the early stages [9]. The Addenbrooke’s Cognitive Examination (ACE-III) was developed as an expansion of the MMSE, but it added more visuospatial, executive, language, and memory tasks [10]. Although the administration time is longer than the other tests, an abbreviated version (Mini-Addenbrooke’s Cognitive Examination, M-ACE) seems to have greater sensitivity for screening [11]. The Rowland Universal Dementia Assessment (RUDAS) has some advantages due to its favorable cross-cultural properties and low susceptibility to the educational level [12,13]. Finally, the Memory Impairment Screen (MIS) is focused on the assessment of episodic memory and, given its brevity, has been suggested for screening uses in primary care [14,15,16]. Nevertheless, even though these tests have been previously validated in different languages and clinical contexts, especially in comparison to the MMSE, as far as we know, few investigations have directly assessed the diagnostic capacity of multiple cognitive screening tests within the same study [17].

In this study, we aimed to evaluate and compare the diagnostic capacity of five cognitive screening tests (ACE-III and M-ACE, MIS, MMSE, MoCA, and RUDAS) for the diagnosis of MCI in patients consulting by memory loss.

## 2. Materials and Methods

### 2.1. Study Design and Participants

We conducted a cross-sectional investigation including patients consulting by memory loss to the Department of Neurology of a tertiary-care hospital in Madrid, Spain. The research protocol was approved by the Ethics Committee of our center. Written informed consent was obtained from all participants.

The patients met the following inclusion criteria: (a) cognitive symptoms mainly focused on memory complaints referred by the patients and/or confirmed by an informant; (b) absence of significant functional impairment according to the Functional Activities Questionnaire (FAQ) [18]; (c) absence of dementia; (d) Spanish-native speaker. The exclusion criteria were as follows: (a) neurological, systemic, or psychiatric disorder potentially impacting cognitive performance or associated with cognitive impairment (e.g., history of stroke, epilepsy, major depression, etc.); (b) visual or auditory impairment that may impair test performance.

The participants were consecutively enrolled among those patients referred for neuropsychological assessment in two periods: September 2021–March 2022 and September 2023–March 2024. Patients were referred to neuropsychological assessment after a consultation by a neurologist, who assessed clinical data reported by the patient and family. No cognitive instruments were administered in this consultation.

Patients underwent a comprehensive neuropsychological examination which included the following tests: verbal span (forward and backward) [19], Corsi block-tapping test (forward and backward) [20], Trail Making Test [21], Symbol Digit Modalities Test [22], Boston Naming Test [23], Free and Cued Selective Reminding Test (FCSRT) [24], Rey-Osterrieth Complex Figure (copy and memory at 3 and 30 min, and recognition) [25], Visual Object and Space Perception Battery (subtests object decision, progressive silhouettes, position discrimination, and number location) [26], Judgment of Line Orientation [27], Stroop Color-Word Interference test [28], and Tower of London-Drexel version [29]. These tests belong to a neuropsychological battery co-normed and validated for the Spanish population in our setting [30,31,32]. Neuropsychological assessment was conducted by trained neuropsychologists.

The total sample comprised 140 participants, 87 (62.14%) women, with a mean age of 74.42 ± 7.60 years and 9.70 ± 5.24 years of formal education. MCI was defined according to one of the following criteria: (a) at least one of the scores of the FCSRT measuring learning or delayed recall (total free recall, total recall, delayed recall) below seven age- and education-adjusted scaled scores (corresponding to >1 SD below the norms); (b) at least two impaired scores (>1 SD below the norms) within the same cognitive domain. These criteria are based on the Jack/Bondi criteria [33] but emphasize the importance of FCSRT in the early diagnosis due to the higher sensitivity of this test to capture the early stages of prodromal AD [34,35]. Accordingly, the patients were classified as cognitively unimpaired (CU) (n = 84) or MCI (n = 56).

The main clinical and demographic characteristics of each group are presented in Table 1.

### 2.2. Cognitive Screening Assessments

Five cognitive screening assessments were administered during a single session, with the sequence of administration changed every thirty cases. Tasks that overlapped across different cognitive tests (such as drawing a clock or specific orientation questions) were presented only once. The following tests were conducted in their Spanish versions: ACE-III [36,37], MIS [38], MMSE [39], MoCA [40], and RUDAS [41].

Normative data were available for all these tests in our specific setting [38,39,42,43,44]. Raw data for all the tests were adjusted for age and education, following the procedures specified for each test, and scaled scores were calculated for ACE-III and MoCA, while corrected scores were computed for MMSE and MIS. For the RUDAS, z-scores were calculated.

**Figure 1:**
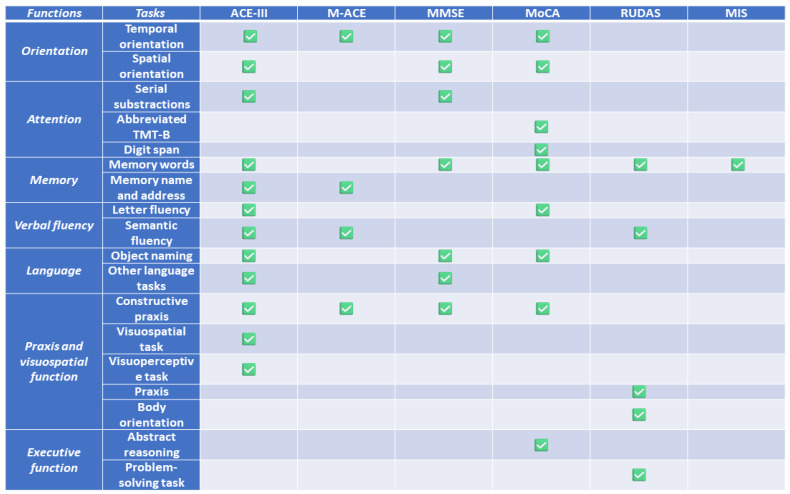
Summary of the main cognitive functions and tasks assessed by each cognitive screening test.

These tests were selected based on the availability of a Spanish version, the use in our setting, and the previous literature suggesting their use in MCI. A summary of the main cognitive functions assessed in each test is depicted in Figure 1.

The time of administration for each test was as follows: 15 min for ACE-III, 5 min for M-ACE, 10 min for MMSE, 10 min for MoCA, 10 min for RUDAS, and 5 min for MIS. The five cognitive tests showed favorable psychometric properties according to previous studies, with high internal consistency. According to Cronbach’s alpha, the internal reliability was 0.92 for ACE-III [10], 0.610 for MMSE [45], 0.83 for M-ACE [11], 0.88 for MoCA [9], and 0.876 for RUDAS [46]. The inter-rater and test-retest reliability was high for all tests [9,10,11,45,46].

### 2.3. Sample Size Estimation

To determine the sample size necessary for comparing two ROC curves using DeLong’s method [47], we calculated first the variance of the difference between the AUC from the 95% intervals of the AUCs. According to our previous study comparing the different cognitive tests in AD dementia [48], it was less than 0.005 in all cases. Then, we calculated that a sample size of 140 individuals would detect a minimum significant difference of 0.0167 between the AUC, assuming a significance level of 0.05 and a statistical power of 0.80.

### 2.4. Statistical Analysis

Statistical analysis was conducted using IBM^®^SPSS Statistics 26.0, Jamovi 2.3.21, and R3.6.3 (pROC package). The results are presented as frequencies (percentages) or mean ± standard deviation. Comparisons were made using Student’s *t*-test (for independent samples) or chi-square tests as appropriate. Cronbach’s alpha was used to estimate the internal consistency of each test. This parameter was interpreted as quite reliable (0.41–0.60), reliable (0.61–0.80), and very reliable (0.81–1.00). Receiver operating characteristic (ROC) curves and the areas under the curves (AUC) were employed to assess the discriminatory ability of each test between MCI and the control group (i.e., patients consulting due to cognitive symptoms but with the standardized neuropsychological assessment battery within normal limits). The method by DeLong et al. was used to compare the ROC curves [47]. ROC curves were generated for both raw scores and adjusted scores using normative data. Youden’s index J was applied to determine the optimal cut-off points. Cohen’s d was calculated to determine effect size, while Pearson’s coefficient was used to measure correlations between tests and between memory scores and the FCSRT. Effect sizes were classified as small (d = 0.2–0.49), moderate (d = 0.5–0.79), and large (d ≥ 0.8). Correlation coefficients <0.40 were interpreted as weak correlations, 0.40–0.69 as moderate, and >0.69 as strong. A *p*-value < 0.05 was considered statistically significant.

Additionally, we estimated the diagnostic capacity of each cognitive test adjusted by the time of administration, as a measure of the efficiency of each tool. Accordingly, we divided the AUC of each total score by the mean time of administration of each test according to the literature [48]. It quantifies how much the diagnostic capacity increases per minute of test administration.

## 3. Results

### 3.1. Diagnostic Validity and Reliability

Patients with MCI showed lower scores in all the cognitive screening tests compared to the control group. Effect sizes were large in M-ACE followed by ACE-III, MMSE, MoCA, and RUDAS, and moderate in MIS (Table 1).

**Figure 2:**
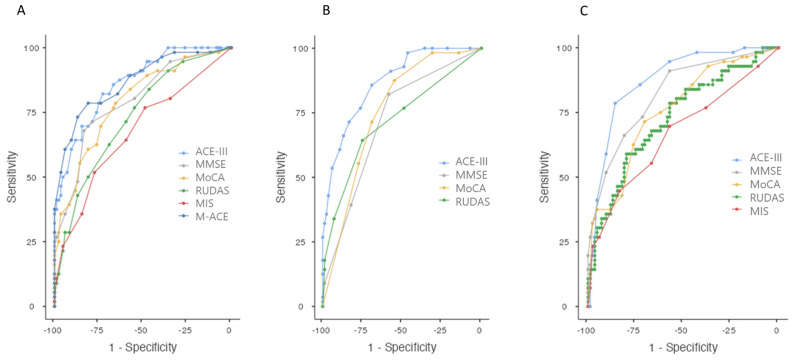
ROC curve analysis for the discrimination between MCI and CU. (**A**) Using total raw scores; (**B**) Using memory scores; (**C**) Using total adjusted scores. ACE-III is shown in blue (M-ACE in dark blue), MMSE in grey, MoCA in yellow, RUDAS in green, and MIS in red.

For the total scores, the AUC was 0.861 (0.802–0.921, 95% confidence interval) for ACE-III, 0.867 (0.805–0.929) for M-ACE, 0.791 (0.715–0.867) for MoCA, 0.795 (0.719–0.872) for MMSE, 0.731 (0.648–0.815) for RUDAS, and 0.672 (0.582–0.762) for MIS. All the ROC curves were statistically significant (*p* < 0.001) (Figure 2A).

The AUC for ACE-III was greater than the AUC of MMSE (*p* = 0.0234), MoCA (*p* < 0.001), RUDAS (*p* < 0.001), and MIS (*p* < 0.001). The AUC for M-ACE was greater than the AUC of MMSE (*p* = 0.03), MoCA (*p* = 0.003), RUDAS (*p* < 0.001) and MIS (*p* < 0.001). The AUC of MoCA was greater than the AUC of MIS (*p* = 0.006). There were no statistically significant differences in the following ROC curve comparisons: M-ACE vs. ACE-III (*p* = 0.755), MoCA vs. MMSE (*p* = 0.891), MoCA vs. RUDAS (*p* = 0.0782), MMSE vs. RUDAS (*p* = 0.088), and MIS vs. RUDAS (*p* = 0.216). The optimal cut-off points, according to the Youden’s index, are shown in Table 2.

For the memory components, the AUC was 0.869 (0.812–0.927) for ACE-III, 0.717 (0.630–0.803) for MMSE, 0.755 (0.676–0.835) for MoCA, and 0.720 (0.630–0.810) for RUDAS. All the ROC curves were statistically significant (*p* < 0.001) (Figure 2B). The AUC of ACE-III (memory) was greater than the AUC of MMSE (*p* < 0.001), MoCA (*p* < 0.001), and RUDAS (*p* < 0.001). There were no statistically significant differences in the following ROC curve comparisons: MMSE vs. MoCA (*p* = 0.381), MMSE vs. RUDAS (*p* = 0.938), and MoCA vs. RUDAS. (*p* = 0.442). The optimal cut-off points, according to the Youden’s index, are shown in Table 2.

For the adjusted scores by age and education, the AUC was 0.875 (0.816–0.934) for ACE-III, 0.822 (0.751–0.893) for MMSE, 0.758 (0.677–0.839) for MoCA, 0.667 (0.573–0.762) for MIS, and 0.729 (0.643–0.815) for RUDAS. All the ROC curves were statistically significant (*p* < 0.001) (Figure 2C). The AUC of ACE-III (*p* < 0.001) and MMSE (*p* = 0.023) were greater than RUDAS (all *p* < 0.001). The AUC of ACE-III was greater than MoCA (*p* = 0.001) and MIS (*p* < 0.001). The AUC of MMSE was greater than MIS (*p* = 0.001). There were no statistically significant differences in the following ROC curve comparisons: ACE-III vs. MMSE (*p* = 0.084), MMSE vs. MoCA (*p* = 0.068), MoCA vs. MIS (*p* = 0.054), MoCA vs. RUDAS (*p* = 0.518), MIS vs. RUDAS (*p* = 0.227). The optimal cut-off points, according to the Youden’s index, are shown in Table 2.

Regarding reliability, the Cronbach’s alpha was 0.827 for ACE-III, 0.505 for MMSE, 0.896 for MoCA, and 0.721 for RUDAS.

### 3.2. Correlations between Tests

Correlations between the total scores of the tests were all strong, except between the MIS and the other tests, which were moderate. All the correlations between the memory scores were moderate, except between MIS and MMSE (memory score), which was weak. All the correlations were statistically significant (Table 3).

The correlation between ACE-III and FCSRT (free total recall, total recall, delayed free recall, and delayed total recall) was 0.796, 0.754, 0.743, and 0.703, respectively. The correlation between M-ACE and FCSRT was 0.805, 0.809, 0.773, and 0.743. The correlation between MMSE and FCSRT was 0.665, 0.683, 0.604, and 0.621, respectively. The correlation between MoCA and FCSRT was 0.77, 0.742, 0.716, and 0.685. The correlation between RUDAS and FCSRT was 0.718, 0.712, 0.705, and 0.673. Finally, the correlation between MIS and FCSRT was 0.629, 0.65, 0.555, and 0.593. All correlations were statistically significant (<0.001).

### 3.3. Efficiency of Each Cognitive Test

The AUC divided per minute of test administration was 0.057 (0.053–0.061) for ACE-III, 0.173 (0.161–0.185) for M-ACE, 0.079 (0.071–0.086) for MoCA, 0.0795 (0.071–0.087) for MMSE, 0.073 (0.064–0.081) for RUDAS, and 0.134 (0.116–0.152) for MIS.

## 4. Discussion

Our study showed that the five cognitive tests distinguished between patients with MCI and the cognitively unimpaired, according to a diagnosis of MCI based on a comprehensive and co-normed neuropsychological battery. This finding is consistent with previous studies validating each individual test [7,8]. However, the main aim of the study and the most interesting finding was the comparison between the different screening tests. In this regard, the ACE-III and its brief version (M-ACE) showed greater diagnostic accuracy than the other cognitive tests, including MoCA, MMSE, MIS, and RUDAS. As M-ACE may be administered in only 5 min [49], our findings suggest that the M-ACE should be the best option considering time of assessment and diagnostic accuracy to screen patients with MCI consulting due to memory loss. In this regard, we calculated an index to measure the increase in AUC per minute of administration time for each cognitive test, confirming that the M-ACE is the most appropriate test for screening, considering the balance between diagnostic capacity and time. This observation may be justified by the fact that the M-ACE only includes the most sensitive items in the early stages of MCI (i.e., temporal orientation, semantic fluency, verbal memory task, clock drawing), whereas the other tests include other items that, although informative about other cognitive domains, are impaired in later stages or in atypical cases (e.g., constructive praxis, naming) [50] (Figure 1). Additionally, the ACE-III provides a more complete assessment of cognitive functions than the other cognitive screening assessments.

As expected, the AUC of each test was reduced compared with a similar study in which we used the same tests for the discrimination between healthy controls and patients with mild dementia [48]. However, the AUC values are still noteworthy and warrant the use of screening cognitive tests for the assessment of patients with memory complaints with no functional impairment. Another important finding is the assessment of reliability. In this regard, reliability was especially high for the ACE-III, good for MoCA and RUDAS, and low for MMSE.

The best cut-off points were estimated according to the Youden’s index, which tries to maximize sensitivity and specificity. The optimal cut-off points estimated for the other tests are the same or similar to those proposed in other studies validating each test in our setting [16]. Regarding the MMSE, the best cut-off was 26 or 29, which is relatively high and seems to confirm previous studies suggesting that MMSE is not sensitive enough to the early stages [51]. However, the choice of cut-off point can also be adapted to the specific setting, according to the clinical needs of higher sensitivity, higher specificity, or predictive values.

Another interesting result is the correlation between the cognitive screening tests with the FCSRT. This test was selected because it is specifically recommended for the diagnosis of early stages of AD [52]. Correlations were higher for M-ACE and ACE-III followed by MoCA and the other tests. These correlations may be interpreted as some sort of concurrent validity of cognitive screening tests, due to the high diagnostic capacity of FCSRT [53].

Our findings have important implications for clinical practice. Memory complaints are a frequent cause of medical consultation in the elderly population and are frequent symptoms in the general population. The early diagnosis and treatment of early stages of AD and other neurodegenerative disorders are increasingly necessary, as MCI patients are at risk of clinical progression. Our findings suggest the superiority of ACE-III and M-ACE over the other cognitive tests in detecting patients with MCI.

Importantly, when considering the time of administration, M-ACE is the most efficient test, whereas other tests like MoCA, MMSE, and RUDAS become more efficient than the ACE-III. This introduces the time of administration as a relevant parameter in selecting the most appropriate cognitive tool, which could be adapted according to different clinical scenarios and the available time to assess each patient. The search for brief tests, considering the time of administration and the associated economic costs, may be especially relevant when implementing modifications in clinical protocols [54]. Additionally, the new landscape of pathology-modifying therapies necessitates optimizing available resources to limit the costs associated with screening, diagnosis, and follow-up [55]. In this regard, our study evaluated the diagnostic capacity of several tests. However, the simultaneous or sequential administration of several brief cognitive tests [56], the incorporation of technological solutions [57,58,59], or the combination between a cognitive tool with a biological biomarker may be of great interest to increase diagnostic accuracy [60] and should be investigated in future investigations about cognitive screening tools.

Our study has some limitations. First, the MoCA optional items for memory were not administered and the MoCA-Memory Index Score was not calculated [61]. Similarly, the MoCA has shown low performances in other Spanish cohorts, which could suggest limitations in the adaptation of the test to the Spanish population that could not be present in other versions [39,50]. Second, the M-ACE was calculated after the administration of the ACE-III, but it was not administered specifically. This could imply a higher diagnostic performance because the time from the registration and recall of the memory task is longer in the ACE-III than in the M-ACE. Third, our study was performed in a tertiary center, although with direct access from primary care. Thus, the PPV and NPV are calculated for this sample, in which the prevalence of MCI was relatively high (40%). Fourth, our study was performed on Spanish-speaking patients to avoid cultural issues in the definition of MCI according to the neuropsychological battery. Future studies should evaluate the comparative diagnostic performance of cognitive screening instruments in multicultural settings [62].

## 5. Conclusions

In conclusion, our study found that the ACE-III and its brief version M-ACE showed better diagnostic properties for the diagnosis of MCI than the other screening tests. MoCA and MMSE showed adequate properties, while the diagnostic capacity of MIS and RUDAS was limited. The M-ACE showed the best balance between diagnostic capacity and time of administration. Future studies combining screening tests and other accessible biomarkers (e.g., plasma biomarkers) would be necessary to define the best strategy to identify patients at risk of developing dementia.

## Figures and Tables

**Table 1:** Main characteristics of the sample.

	CU	MCI	Statistic (*p*-Value)	Cohen’s d
Number of subjects N	84	56	-	-
Age	74.06 ± 8.64	74.98 ± 5.73	−0.702 (0.484)	-
Sex (females) N (%)	56 (66.7%)	31 (55.4%)		-
Years of education	10.05 ± 5.35	9.20 ± 5.06	0.941 (0.348)	-
Country of birth	81 (96.4%) Spain 3 (3.6%) Latin America	55 (98.2%) Spain 1 (1.8%) Latin America	0.386 (0.534)	-
ACE-III	84.30 ± 9.43	66.75 ± 13.38	9.100 (<0.001)	1.570
M-ACE	23.99 ± 3.70	17.02 ± 4.92	9.554 (<0.001)	1.648
MIS	5.99 ± 1.96	4.55 ± 2.36	3.895 (<0.001)	0.672
MMSE	28.30 ± 1.88	24.98 ± 3.45	7.323 (<0.001)	1.263
MoCA	23.43 ± 4.78	17.23 ± 5.79	6.895 (<0.001)	1.190
RUDAS	26.38 ± 3.37	22.89 ± 4.61	5.162 (<0.001)	0.891

**Table 2:** Optimal cut-off points, sensitivity, specificity, predictive values, and Youden index of each test.

	Optimal Cut-Off	Youden Index J	Sensitivity	Specificity	PPV	NPV
Total raw scores						
ACE-III	78	0.548	82.14%	72.62%	66.69%	84%
Mini-ACE	20	0.601	78.57%	80.95%	73.33%	85%
MIS	5	0.292	51.79%	77.38%	60.42%	70.65%
MMSE	26	0.512	67.86%	83.33%	73.08%	79.55%
MoCA	22	0.440	78.57%	65.48%	60.27%	82.09%
RUDAS	13	0.315	62.50%	69.05%	57.38%	73.42%
Adjusted scores						
ACE-III *	8	0.643	78.57%	85.71%	78.57%	85.71%
MMSE	29	0.482	91.07%	57.14%	58.62%	90.57%
MoCA *	7	0.417	71.43%	70.24%	61.54%	78.67%
MIS	4	0.280	44.64%	83.33%	64.1%	69.31%
RUDAS *	−1.59	0.375	58.93%	79.76%	66%	74.44%
Memory scores						
ACE-III	14	0.548	85.71%	69.05%	64.86%	87.88%
MMSE	3	0.405	82.14%	58.33%	56.79%	83.05%
MoCA	3	0.423	87.5%	54.76%	56.32%	86.79%
RUDAS	4	0.393	64.29%	75%	63.16%	75.9%

* Adjusted scores are shown in scaled scores for ACE-III and MoCA and z-scores for RUDAS.

**Table 3:** Correlations between tests.

		ACE-III	MIS	MMSE	MoCA	RUDAS
		Correlations (Total Scores)				
**ACE-III**	**Correlations (memory scores)**	-	0.507	0.799	0.886	0.720
**MIS**		0.512	-	0.489	0.566	0.485
**MMSE**		0.637	0.352	-	0.764	0.671
**MoCA**		0.640	0.518	0.478	-	0.739
**RUDAS**		0.617	0.510	0.496	0.555	-

## Data Availability

The datasets used and/or analyzed during the current study are available from the corresponding author on reasonable request, due to ethical committee restrictions.
